# Serum vascular endothelial growth factor changes and safety after topical anti‐human VEGF antibody bevacizumab in healthy dogs

**DOI:** 10.1111/vop.12628

**Published:** 2019-02-04

**Authors:** Lisa‐Marie Muellerleile, Alexander Tichy, Barbara Nell

**Affiliations:** ^1^ Department of Companion Animals and Horses University of Veterinary Medicine Vienna Vienna Austria; ^2^ Department of Biomedical Sciences University of Veterinary Medicine Vienna Vienna Austria

**Keywords:** bevacizumab, dog, safety, VEGF

## Abstract

**Objective:**

To evaluate ocular and general safety of topical anti‐human VEGF bevacizumab and the effect on serum vascular endothelial growth factor (VEGF) values in healthy dogs.

**Procedures:**

Nine university‐owned beagles received 0.05 mL of 0.25% bevacizumab eyedrops (Avastin^®^, Roche) in one eye and 0.05 mL of 0.9% saline solution in the other eye as a control, administered at 12 hours intervals over a period of 28 days. Continuous monitoring for vital parameters and ocular examinations were conducted. Complete blood counts including hematology and coagulation parameters were performed before trial start as well as 24 hours, 7 days, and 28 days after trial start. Measurements of serum VEGF values were obtained using an ELISA‐based approach at days 0, 7, and 28. The experiment was designed as a masked placebo‐controlled study.

**Results:**

No clinical signs of ocular toxicity or systemic incompatibility were noted in any dog at any time point of the study. No signs of pain were present in any dog at any time point. All blood count values remained in normal clinical ranges without relevant variation. There was no significant change in mean serum VEGF values between day 0 and day 7 and between day 0 and day 28.

**Conclusions:**

The results indicate that topical bevacizumab treatment is safe in healthy dogs. However, further studies are needed to assess safety and efficacy in diseased dogs with naturally occurring corneal neovascularization.

## 1 INTRODUCTION

Corneal neovascularization (CNV) and corneal opacification arise concomitantly in several corneal diseases in dogs, for instance keratoconjunctivitis sicca, superficial pigmentary keratitis, and chronic superficial keratitis.^1^, ^2^, ^3^ Although corneal neovascularization is initially essential for corneal wound healing and hindering stromal melting, corneal neovascularization can cause blindness, tissue scarring, lipid deposition, edema, and potentially sustains inflammation.^4^, ^5^ Furthermore, avascularity is one of the prerequisites of the corneal immune privilege.^6^ Hence, inhibition of corneal neovascularization represents a therapeutic strategy in animals with chronic keratitis and CNV. Promising therapeutic results in animal models and human patients with CNV have been reported for anti‐human vascular endothelial growth factor (VEGF) therapies.^7^, ^8^, ^9^, ^10^

VEGF‐A is a glycosylated endothelial mitogen that is involved in pathological angiogenesis and is known to be increased in inflamed and vascularized corneas.^11^, ^12^, ^13^ Hence, pharmacological inhibition of VEGF‐A is a promising strategy to treat diseases driven by pathological neovascularization. There is an arsenal of distinct VEGF inhibitors differing in their antiangiogenetic activity, binding affinity for VEGF‐A, and their binding spectrum to different VEGF isoforms and other members of the VEGF family.^14^ One of the most potent VEGF inhibitors is bevacizumab. In human ophthalmology, an off‐label intravitreal use of bevacizumab is already taking place and is reported to be effective for the treatment of wet age‐related macular degeneration, diabetic macular edema, macular edema secondary to retinal vein occlusion, vascular glaucoma, and CNV.^7^, ^8^, ^15^, ^16^, ^17^

So far no investigations have been carried out to determine the safety and medical compatibility of topical bevacizumab in dogs. There are several animal models which have proven the safety and efficacy of subconjunctivally and topically administered bevacizumab in mice, rabbits, and chinchilla bastard rabbits.^10^, ^18^, ^19^ It has been shown that topical bevacizumab treatment does not have a significant influence on corneal integrity, corneal wound repair, and corneal nerve fiber density in mice with experimentally induced corneal epithelial abrasions.^10^, ^18^, ^19^, ^20^, ^21^

Systemic side effects have been reported after intravitreal injections of bevacizumab in humans, such as systemic hypertension, cerebrovascular accidents, and facial skin redness.^22^, ^23^ A decrease of plasma VEGF values after intravitreal bevacizumab injections has been described.^24^ However, there is no knowledge about effects on serum VEGF values and general safety after topically applied bevacizumab in dogs.

The aim of the current work was to investigate the safety and medical compatibility of topical administered bevacizumab in healthy dogs. Additionally, we studied the effect of topical bevacizumab treatment on serum VEGF values in healthy dogs. We hypothesized that topical administered bevacizumab is both systemically and topically safe and has no effect on serum VEGF values.

## 2 MATERIAL AND METHODS

### 2.1 Animals

The study was approved by the institutional ethics and animal welfare committee and the national authority according to §§ 26ff. of Animal Experiments Act, Tierversuchsgesetz 2012 —TVG 2012 (GZ 68.205/0134‐WF/V/3b/2017). The trial was conducted on 10 university‐owned beagle dogs aged at least 16 months and bred for experimental purpose. The experiment was designed as a masked placebo‐controlled study.

Each dog underwent a full physical and ocular examination to ensure eligibility requirements prior to initiation of the study. The arterial blood pressure was measured and a complete blood count including hematology, baseline serum VEGF values, activated partial thromboplastin time, partial thromboplastin time, and thrombin time was conducted. A complete ophthalmic examination was performed in all dogs by the first author (LM) under supervision of a board‐certified ophthalmologist (BN). The examination included slit‐lamp biomicroscopy (Kowa SL‐15; Kowa, Tokyo, Japan), indirect ophthalmoscopy (Keeler Vantage; Keeler Instruments Inc, Broomall, PA), Schirmer tear test‐1 (Teststreifen, MSD, Unterschleissheim, Germany), fluorescein staining (Fluorotouch Ophthalmic Strips, Eickemeyer, Tuttlingen, Germany), and intraocular pressure measurements using rebound tonometry (TonoVet, Icare, Vantaa, Finland). Posterior segment examination was conducted after pharmacological mydriasis (Mydriaticum, Agepha, Senec, Slovakia). Dogs had to be free of any systemic and ocular disease and did not receive ocular drugs, any kind of systemic medication, VEGF affecting drugs or agents consisting of human proteins 14 days before the start of the trial.

### 2.2 Drug preparation

A 0.25% solution of bevacizumab eyedrops was aseptically prepared from a commercial available intravenous bevacizumab solution (Avastin^®^, Roche, Basel, Switzerland) by the institute's pharmacy in compliance with good manufacturing practice. Sterile 0.9% saline solution was used as the solvent, and the study drug was stored at 2‐8°C and protected from light with a durability of 28 days.^25^ Sterile 0.9% saline solution served as the control.

The pharmacist prepared single‐dose containers for each dog with a single dose of either the study medication (0.05 mL of 0.25% bevacizumab solution) or the placebo (0.05 mL of 0.9% saline solution). The containers were labeled with the information “left eye” or “right eye” and only the pharmacist had knowledge about the content. After the trial, the pharmacist revealed which eye received bevacizumab or placebo.

### 2.3 Study design

All examinations were performed by the same experienced observer (LM). Beagle dogs received 0.05 mL of 0.25% bevacizumab eyedrops in one eye and 0.05 mL of 0.9% saline solution in the other eye as a control, administered at 12 hours intervals over a period of 28 days.

Continuous monitoring was scheduled as follows: Over a period of 3 hours after the first drug administration monitoring of vital parameters (including respiratory and heart rate, mucous membrane color, capillary refill time, and arterial blood pressure) was performed hourly. Thereafter, the parameters were taken every 3 hours for the duration of 24 hours after trial start. At day 2‐7, dogs underwent a full physical examination and arterial blood pressure measurements once a day. The final examination was performed at day 28 after trial start.

Complete blood counts including hematology and coagulation parameters (activated partial thromboplastin time, partial thromboplastin time, and thrombin time) were performed 24 hours, 7 days, and 28 days after trial start. Venous blood sample was obtained via the cephalic vein.

Indirect blood pressure measurements were obtained by using an oscillometric device (PETMAP™ graph II, Ramsey Medical, USA, Tampa). Dogs were allowed to settle for several minutes before the blood pressure readings began. An appropriate cuff (Critter Cuff™, Ramsey Medical, USA, Tampa) with a width of about 40% of the forelimb circumference was applied and linked to the oscillometric pressure unit. Readings were taken from the median artery by putting the cuff around the right midradial region overlying the median artery. Dogs were restrained gently on the examination table in sternal recumbency, placing the cuff at the level of the right heart. For each dog, three readings (each systolic, diastolic, and mean arterial pressure) were taken successively.

Ophthalmic examinations were performed directly after drug administration, as well as 3 hours, 24 hours, once a day at day 2‐7, and at day 28. They were performed without pharmacological mydriasis except the final examination at day 28. A modified Hackett‐McDonald scoring system^26^ and a pain score system^27^ were used to determine ocular and systemic toxicity (see below).

### 2.4 Ocular irritation and assessment of ocular toxicity potential

Ocular examination findings were scored using a modified Hackett‐McDonald scoring system.^26^ Examination scores were recorded for conjunctival congestion/hyperemia (0‐3+), conjunctival chemosis/swelling (0‐3+), conjunctival/ocular discharge (0‐3+), corneal edema (0‐3+), and corneal vascularization (0‐3+). Moreover, all dogs were assessed using a subjective pain scoring system^27^: Pain scoring categories included comfort, movement, degree of blepharospasm, unprovoked behavior, interactive behaviors, and vocalization (Table 1).

**Table 1:** Pain score system modified from Clark et al^27^ to determine ocular and systemic toxicity in healthy dogs after topical bevacizumab application

Pain scoring category	Manifestation
Comfort	0 = dog is calm and interested in surroundings
	1 = dog shows mild agitation or is depressed, not interested in surroundings
	2 = dog shows moderate agitation and is restless
	3 = dog is extremely agitated
Movement	0 = dog is quiet
	1 = about 1‐2 position changes per minute
	2 = about 2‐6 position changes per minute
	3 = dog shows continuous changes
Appearance/blepharospasm	0 = eyelids are completely open and in physiological position
	1 = eyelids are partially closed (ca. 25%)
	2 = eyelids are partially closed (ca. 50%); dog shows mild tearing
	3 = eyelids are partially closed (ca. 75%); dog shows moderate tearing
	4 = eyelids are completely closed; dog shows marked tearing
Behavior (unprovoked)	0 = normal
	1 = minor changes
	2 = moderately abnormal (less mobile or alert than normal, not interested in surroundings, fretful)
	3 = noticeably abnormal (fretful, vocalizing, self‐mutilation, groaning)
Interactive behaviors	0 = normal
	1 = pulls head away when eyes getting touched
	2 = vocalizes when eyes getting touched
	3 = violent reaction to touching of eye (biting, snapping, groaning)
Vocalization	0 = quiet
	1 = dog cries but responds to be quiet
	2 = dog intermittently cries without response to quiet voice
	3 = dog constantly cries without response to quiet voice

### 2.5 Serum VEGF values: Enzyme‐linked Immunosorbent Assay

Serum samples were centrifuged for 20 minutes at 1000× g within 30 minutes of collection. Afterward, samples were stored in sterile polypropylene tubes at ≤−80°C. Measurement of serum VEGF values was obtained using an ELISA‐based approach. For that a commercially available canine enzyme‐linked immunosorbent assay was used (Canine VEGF Quantikine ELISA Kit, R&D Systems, Minneapolis, USA, Cat # CAVE00). VEGF values were measured before, 7 days, and 28 days after trial start. The ELISA was conducted in accordance with the assay instructions. All standards and samples were analyzed in duplicate.

### 2.6 Statistical analysis

The current work was designed as a pilot study. Currently, safety and medical compatibility of topical bevacizumab in dogs are uncharted, and effect sizes and statistical variation are unknown. Sample size planning based upon comparable study schedules of former studies in rodent models.^20^, ^28^

Statistical analysis was performed using the software program SPSS (IBM SPSS Statistics 24).

The clinical endpoint was the occurrence of ocular or systemic adverse events. The frequency and type of systemic and ocular recorded side effects were analyzed in a descriptive manner.

Quantitative data were summarized as mean ± standard deviation. The confidence interval was computed from the observed data, using confidence values of 95%. Furthermore, a paired‐sample *t* test was used to investigate differences of the arterial blood pressure, heart rate, respiratory rate, and coagulation parameters between day 0, day 1, day 7, and day 28. Changes in VEGF serum values were compared between day 0, day 7, and day 28 using a paired‐sample *t* test. The assumption of normal distribution was tested using Kolmogorov‐Smirnov test. A *P*‐value <0.05 was considered as statistically significant.

## 3 RESULTS

### 3.1 Animals

Nine of 10 clinic‐owned beagle dogs were included in the study. One of the beagles was not concordant with the eligibility requirements as he showed a slight thrombocytopenia and thus was excluded from the study. All study dogs were male with a mean weight of 14.7 ± 2.5 kg and the median age was 30 (range 23‐39) months.

### 3.2 Ocular toxicity potential

No clinical signs of ocular toxicity or ocular adverse events such as conjunctival hyperemia or chemosis, ocular discharge, corneal edema, corneal vascularization, or corneal defects were noted in either eye of any dog at any time point of the study. Intraocular pressure measurements and values of Schirmer tear test‐1 values remained within normal limits, with minimal variations without clinical relevance in any dog at any time point. Thus, only the confidence interval and standard deviation was computed and are illustrated in Table 2.

**Table 2:** Mean ± SD of Schirmer tear test (mm/min) and intraocular pressure (mm Hg) in healthy dogs after topical bevacizumab application at baseline and on days 1, 7, and 28 of the study

	Day 0	Day 1	Day 7	Day 28
STT (mm/min)	20.0 ± 2.2	20.6 ± 1.8	21.2 ± 2.3	20.6 ± 1.8
IOP (mm Hg)	18.2 ± 1.0	17.1 ± 1.1	17.4 ± 1.4	17.2 ± 1.2

IOP, intraocular pressure; SD, standard deviation; STT, Schirmer tear test.

No signs suggestive of pain using a subjective pain scoring system were present in any dog at any time point.

### 3.3 Systemic toxicity potential

No clinical signs of systemic incompatibility or adverse events were noted in any dog at any time point. All values remained in normal clinical ranges without relevant variation. Thus, only the confidence interval and standard deviation were computed.

Values of the differential blood count and coagulation parameters remained within the normal range.

There was no significant change in mean serum VEGF values between day 0 and day 7 (50.8 ± 18.6 pg/mL vs 55.8 ± 11.2 pg/mL, respectively; *P* = 0.72) and between day 0 and day 28 (50.8 ± 18.6 pg/mL vs 52.9 ± 17.0 pg/mL, respectively; *P* = 0.47).

All recorded data are shown in Table 3.

**Table 3:** Mean ± SD of heart rate, respiratory rate, systolic and diastolic blood pressure, and serum VEGF levels in healthy dogs after topical bevacizumab application at baseline and on days 1‐7 and day 28 of the study

	Day 0	Day 1	Day 2	Day 3	Day 4	Day 5	Day 6	Day 7	Day 28
Heart rate (heartbeat/minute)	98.7 ± 11	95.6 ± 8.1	94.2 ± 6.0	92.9 ± 4.8	97.3 ± 8.7	96.4 ± 7.6	94.2 ± 7.0	95.6 ± 8.1	94.2 ± 4.5
Respiratory rate (breaths/minute)	23.6 ± 4.2	24.0 ± 2.8	23.1 ± 3.3	23.6 ± 2.4	23.6 ± 3.1	23.6 ± 2.4	22.2 ± 2.9	23.1 ± 3.3	24.0 ± 2.8
BP (systolic) (mm Hg)	147.1 ± 5.5	143.4 ± 3.4	142.7 ± 4.4	141.6 ± 3.2	141.8 ± 3.9	144.4 ± 3.9	142.8 ± 3.5	145.6 ± 4.7	148.6 ± 6.6
BP (diastolic) (mm Hg)	73.6 ± 9.6	73.7 ± 7.3	73.6 ± 7.6	72.3 ± 10.2	74.6 ± 8.3	74.0 ± 8.3	74.2 ± 7.8	73.6 ± 8.6	73.1 ± 10.1
Serum VEGF (pg/mL)	50.8 ± 18.6							55.8 ± 11.2	52.9 ± 17.0

BP, blood pressure; SD, standard deviation; VEGF, vascular endothelial growth factor.

## 4 DISCUSSION

In human medicine, there is a widely off‐label use of bevacizumab for the treatment of various eye diseases accompanied by pathological angiogenesis.^7^, ^8^, ^15^, ^16^, ^17^ Most of them are retinal, choroidal, and corneal diseases such as neovascular age‐related macular degeneration (AMD), diabetic macular edema and macular edema secondary to retinal vein occlusion (RVO), and superficial corneal diseases associated with corneal neovascularization. Chronic keratitis and corneal neovascularization are also common in veterinary ophthalmology^1^, ^2^, ^3^ and a high need for target‐directed treatments exists. However, data concerning medical compatibility, safety, and efficacy of bevacizumab in animals are rare.

Abrams et al^29^ found that VEGF is higher in aqueous humor than in plasma of diabetic and nondiabetic cataractous dogs suggesting a local production of VEGF within the eye. This assumption is further supported by the observation, that there is a constitutive expression of VEGF receptor‐1 by endothelial cells and nonvascular cells of the cornea, uvea, lens, and retina of dogs.^30^ It is known that dogs with glaucoma, uveitis, and intraocular neoplasia show a higher VEGF receptor‐2 expression than healthy dogs, suggesting a role of VEGF in pathologic angiogenesis in canine eyes.^30^ This leads us to the assumption that VEGF is also contributing to pathological vascularization in the cornea of dogs and a therapeutic application of anti‐VEGF substances in canine patients with keratitis and CNV is conceivable.

There is an arsenal of various anti‐VEGF substances.^31^ We chose bevacizumab as it is commercially available and fits a cost‐effectiveness ratio suitable for veterinary use.

For the future development of anti‐VEGF treatments in veterinary medicine, it is important to keep in mind that bevacizumab is a humanized murine antibody that is designed to bind human VEGF‐A. Hence, pharmacological suitability of bevacizumab in veterinary patients might be critically questioned. Recent in vitro experiments demonstrated that bevacizumab binds canine VEGF dose‐dependently (Muellerleile et al, submitted to journal 2018). Interestingly, feline and equine VEGF showed linear, dose‐independent binding characteristics, suggesting only a non‐specific interaction in cats and horses (Muellerleile et al, submitted).

Aside from pharmacologically suitability, the safety profile of bevacizumab for possible future clinical applications is important. Medical compatibility and the occurrence of side effects depend on the application type and the drug dose.^14^

In the present study, no ocular and systemic side effect occurred after topical bevacizumab treatment (2.5 mg/mL BID). These results correlate with those previously reported in people and rodent models.^8^, ^20^, ^21^ However, Kim et al described the occurrence of corneal erosions and corneal thinning in people with CNV after long‐term bevacizumab treatment (12.5 mg BID).^18^ It is noteworthy that the frequency and dose of bevacizumab were much higher in this study and diseased people were examined. However, we decided to use clinically relevant and viable doses with respect to possible future applications of bevacizumab.

Systemic side effects of anti‐VEGF therapies have been reported after intravenous and intravitreal bevacizumab injections in human patients. These application types are associated with serious and sometimes fatal side effects, for example, gastrointestinal perforations, hemorrhage, hypertension, cardiac ischemia, cerebrovascular ischemia, and arterial thromboembolic events.^22^, ^23^

It is known that bevacizumab is detectable in serum after intravitreal injections.^24^ These observations lead to the concern that topical bevacizumab has the potential to enter the systemic circulation and thus causing systemic effects. We considered it unlikely as there were no documented side effects after topical bevacizumab treatment. Nevertheless, as a precaution, we investigated systemic effects to estimate a potential systemic reaction after topical treatment.

Furthermore, we tested the effect of topical bevacizumab on serum VEGF values. It was so far unknown if topical bevacizumab treatment leads to serum VEGF changes. This topic has been intensively studied for intravitreal injections.^24^ In those studies, bevacizumab concentrations reached a maximum serum concentration 7 or 8 days after intravitreal injection.^24^ In our study, there was no significant effect on serum VEGF values after topical bevacizumab treatment (2.5 mg/mL BID) over a treatment period of 28 days.

There were some limitations in this study. The sample size was small. Although our findings were favorable, a larger sample size would have gained confidence in the statistics. All analyzed beagles were healthy, male, and relative young. Discrepancies between healthy corneas, corneas of older dogs, and corneas suffering from chronic inflammation are possible due to different corneal VEGF concentrations and structural integrity alterations.

Another shortcoming of our study is that no untreated dogs were included. As safety after topical bevacizumab in healthy dogs was the major question of our study, we decided to use the contralateral eye as control. Baseline blood values and baseline vital parameters before treatment were compared to those after treatment start to detect systemic effects. No systemic side effects are reported in human medicine^8^ and no side effects occurred in our study so one might argue the value of a separated control group. Anyway, we considered that there was no real control group and we confessed that a separation into a control group and a treatment group would have obtained a better reasonable assurance particularly for systemic effects.

Chosen by the institutional pharmacist the left eye was the treated one. One might argue that laterality plays a role and the left eyes could have reacted differently. There is no publication supporting this theory. Bilateral administration might lead to a higher serum drug concentration, however probably not high enough to cause side effects. In human medicine, no systemic side effects have been reported after topical bevacizumab even in higher concentrations and after bilateral use.^8^ However, further studies are required to study larger animal groups with uni‐ and bilaterally affected and treated dogs.

Another limitation of our study is the treatment duration and drug concentration. Currently, there is no knowledge about safety of topical bevacizumab in dogs. In accordance to treatment protocols of former clinical human studies, an approved concentration of 2.5 mg/mL^7^ was tested over a period of 28 days to find a basic idea. Anyway, it would have been interesting to investigate the safety, changes of serum VEGF values, and particularly the effectiveness of bevacizumab in higher drug concentrations. We are aware that patients with chronic keratitis require long‐term therapy and future studies will be necessary to evaluate the effect and safety over a longer period than 28 days.

## 5 CONCLUSION

In summary, topical bevacizumab (2.5 mg/mL BID) seems to be topically and systemically safe in healthy dogs. Additionally, no changes in serum VEGF values after topically administered bevacizumab were observed. Our results provide a basis for the future development of anti‐VEGF treatments for veterinary use. However, further studies are needed to assess differences between uni‐ and bilateral use, laterality, and a longer treatment period. It will be an issue of future studies to investigate the safety and efficacy in diseased dogs with naturally occurring corneal neovascularization.
